# Increased epigenetic age in normal breast tissue from luminal breast cancer patients

**DOI:** 10.1186/s13148-018-0534-8

**Published:** 2018-08-29

**Authors:** Erin W. Hofstatter, Steve Horvath, Disha Dalela, Piyush Gupta, Anees B. Chagpar, Vikram B. Wali, Veerle Bossuyt, Anna Maria Storniolo, Christos Hatzis, Gauri Patwardhan, Marie-Kristin Von Wahlde, Meghan Butler, Lianne Epstein, Karen Stavris, Tracy Sturrock, Alexander Au, Stephanie Kwei, Lajos Pusztai

**Affiliations:** 1 Department of Internal Medicine, Section of Medical Oncology, Yale School of Medicine, 300 George Street, Suite 120, New Haven, CT 06511 USA; 2 Department of Human Genetics, David Geffen School of Medicine, University of California Los Angeles, Los Angeles, CA 90095 USA; 3 Department of Biostatistics, Fielding School of Public Health, University of California Los Angeles, Los Angeles, CA 90095 USA; 4 Department of Pharmacology, Yale School of Medicine, 333 Cedar Street, New Haven, CT 06511 USA; 5 Department of Surgery, Memorial Sloan Kettering Cancer Center, New York, 10065 USA; 6 Department of Surgery, Yale School of Medicine, 330 Cedar Street, New Haven, CT 06511 USA; 7 Department of Pathology, Yale School of Medicine, 330 Cedar Street, New Haven, CT 06511 USA; 8 Department of Internal Medicine, Indiana University Melvin and Bren Simon Cancer Center, Indianapolis, IN 46202 USA; 9 Department of Obstetrics and Gynecology, Münster University Hospital, Münster, Germany; 10 Department of Clinical Surgery, Perelman School of Medicine, University of Pennsylvania, Philadelphia, PA 19104 USA

**Keywords:** Humans, DNA methylation, Genome, Multivariate analysis, Epigenetics, Breast, Epigenomics, Breast neoplasms, Biomarkers, Smoking

## Abstract

**Background:**

Age is one of the most important risk factors for developing breast cancer. However, age-related changes in normal breast tissue that potentially lead to breast cancer are incompletely understood. Quantifying tissue-level DNA methylation can contribute to understanding these processes. We hypothesized that occurrence of breast cancer should be associated with an acceleration of epigenetic aging in normal breast tissue.

**Results:**

Ninety-six normal breast tissue samples were obtained from 88 subjects (breast cancer = 35 subjects/40 samples, unaffected = 53 subjects/53 samples). Normal tissue samples from breast cancer patients were obtained from distant non-tumor sites of primary mastectomy specimens, while samples from unaffected women were obtained from the Komen Tissue Bank (*n* = 25) and from non-cancer-related breast surgery specimens (*n* = 28). Patients were further stratified into four cohorts: age < 50 years with and without breast cancer and age ≥ 50 with and without breast cancer. The Illumina HumanMethylation450k BeadChip microarray was used to generate methylation profiles from extracted DNA samples. Data was analyzed using the “Epigenetic Clock,” a published biomarker of aging based on a defined set of 353 CpGs in the human genome. The resulting age estimate, DNA methylation age, was related to chronological age and to breast cancer status.

The DNAmAge of normal breast tissue was strongly correlated with chronological age (*r* = 0.712, *p* < 0.001). Compared to unaffected peers, breast cancer patients exhibited significant age acceleration in their normal breast tissue (*p* = 0.002). Multivariate analysis revealed that epigenetic age acceleration in the normal breast tissue of subjects with cancer remained significant after adjusting for clinical and demographic variables. Additionally, smoking was found to be positively correlated with epigenetic aging in normal breast tissue (*p* = 0.012).

**Conclusions:**

Women with luminal breast cancer exhibit significant epigenetic age acceleration in normal adjacent breast tissue, which is consistent with an analogous finding in malignant breast tissue. Smoking is also associated with epigenetic age acceleration in normal breast tissue. Further studies are needed to determine whether epigenetic age acceleration in normal breast tissue is predictive of incident breast cancer and whether this mediates the risk of chronological age on breast cancer risk.

**Electronic supplementary material:**

The online version of this article (10.1186/s13148-018-0534-8) contains supplementary material, which is available to authorized users.

## Background

Breast cancer represents 15% of all new cancer cases in the US, and with 252,710 estimated new cases in 2017, it has the highest cancer-related incidence in women in the country [1]. Age is one of the strongest risk factors for developing breast cancer and is most frequently diagnosed among women aged 55 to 64. However, the factors that mediate the effect of chronological age on breast cancer are not fully known. Since epigenetic changes are one of the hallmarks of aging, it is plausible that age-related epigenetic changes may play a role in conferring breast cancer risk.

Historically, studies of the effect of age on breast cancer have been limited by the lack of suitable molecular biomarkers of tissue age. Several studies have explored whether telomere shortening is associated with increased risk and earlier occurrence of familial breast cancer, but the reported effect sizes are relatively weak and require additional validation [2, 3].

It has recently been recognized that DNA methylation levels lend themselves for defining a highly accurate biomarker of tissue age (“epigenetic clock”) that applies to all human tissues and cell types [4]. This epigenetic biomarker is based on the weighted average DNA methylation level of 353 cytosine-phosphate-guanines (CpGs). The age estimate (in unit of years) is referred to as “DNA methylation age” (DNAmAge) or “epigenetic age.” By contrasting DNAmAge with an individual’s chronological age, one can define a measure of epigenetic age acceleration. For instance, a woman whose blood tissue has a higher DNAmAge than expected based upon her chronological age is said to exhibit positive age acceleration in blood. Recent studies support the idea that these measures are at least passive biomarkers of biological age. To elaborate, the epigenetic age of blood has been found to be predictive of all-cause mortality [2, 3], lung cancer [5], frailty [6], and cognitive and physical functioning [7]. Further, the utility of the epigenetic clock method using various tissues and organs has been demonstrated in several applications including Alzheimer’s disease [8], centenarian status [8, 9], obesity [10], menopause [11], and osteoarthritis [12].

An increasing body of literature suggests that epigenetic age acceleration in blood is predictive of various cancers [5, 13] including breast cancer [14]. Cancer greatly disrupts the epigenetic age of the affected (malignant) tissue [4, 15]. While some cancer types are associated with positive age acceleration, others are associated with negative age acceleration [4, 15]. We have recently shown that *malignant* breast cancer samples from luminal breast cancer exhibit strong positive age acceleration, which contrasts sharply with the negative age acceleration in basal breast cancers [4, 15]. However, it is unknown whether these age acceleration effects can also be observed in a *normal* adjacent tissue. Here, we address this question by correlating epigenetic age acceleration in normal breast tissue samples with breast cancer disease status. We find that normal breast tissue samples from breast cancer cases exhibit positive age acceleration compared to normal breast tissue samples from controls. These age acceleration effects are independent of various confounders such as chronological age, ethnicity, age at menarche, number of live births, and menstrual status. In a secondary analysis, we found that smoking is associated with positive epigenetic age acceleration in normal breast tissue.

## Methods

### Study specimens

This was a multicenter cross-sectional study performed on fresh frozen samples of normal breast tissue that were collected from four cohorts of women, namely age < 50 years with and without breast cancer and age ≥ 50 with and without breast cancer. Normal breast tissue in patients with breast cancer was defined as histologically benign tissue at least 3 cm away from the primary tumor margin. These samples were obtained prospectively from patients undergoing primary total mastectomy for stage 0–III breast cancer at the Yale Breast Center. Eligible patients were those who had not received chemotherapy, radiation, or endocrine therapy prior to surgery. Normal breast tissue from non-cancer patients was obtained from the Susan G. Komen Tissue Bank at IU Simon Cancer Center and prospectively from women presenting for reduction mammoplasty at Yale New Haven Hospital. Clinical data collected for each subject included age, height, weight, ethnicity, medical history, reproductive history, tobacco and alcohol use, family history of breast cancer, and tumor characteristics. The study was approved by the institutional review board, and written informed consent was obtained from all patients in compliance with the protocol.

The Susan G. Komen Tissue Bank (KTB) is a unique resource that has helped in the understanding of normal breast biology. All participant samples from the KTB group are unaffected tissue donors without a cancer history, and study samples were anonymized in accordance to the protocol. The study population from the hospital prospective cohort included women from all age groups that consented for the study, and patients that had received neoadjuvant treatment were excluded. The tissue samples were further categorized based on tumor molecular subtypes.

### Tissue processing

The breast tissue was sampled as six individual core pieces that were histologically benign, and within 5 min of procurement, each piece was embedded in a cassette that was subsequently placed in a 10% buffered formalin solution and stored at room temperature. The cores were then flash frozen with liquid nitrogen at − 166.2 °C. The cryo-vials with at least 50 mg of breast tissue per sample were shipped to the lab where the DNA was extracted using the Qiagen All Prep Universal kit. Samples were processed as whole tissue, and DNA was re-extracted from samples that had low DNA yield because of increased fatty tissue. The extracted DNA was then used for bisulfite sequencing experiments.

### DNA extraction and methylation studies

Zymo EZ DNA methylation KIT (Zymo Research, Orange, CA, USA) was used to obtain bisulfite conversion and subsequent hybridization, and scanning was performed with the HumanMethylation450k BeadChip (Illumina, San Diego, CA) and iScan (Illumina) according to the manufacturers’ protocol with standard settings. DNA methylation levels (*β*) were quantified using the “noob” normalization method [16]. Specifically, the *β* value was calculated as a ratio of the intensity of fluorescent signals from the methylated and the unmethylated sites:

*β* = max (*M*,0)/[max (*M*,0) + max (*U*,0) + 100].

*M* = methylated signals.

*U* = unmethylated signal.

Thus, *β* values ranged from 0 to 1 (completely unmethylated to completely methylated).

DNAmAge was then calculated, which has been described in detail elsewhere [4]. Briefly, the epigenetic clock is defined as a prediction method of age based on the DNA methylation levels of 353 CpGs. Predicted age, referred to as DNAmAge, correlates with chronological age in multiple different cell types (CD4+ T cells, monocytes, B cells, neurons), tissues, and organs, including whole blood, brain, breast, kidney, liver, and lung [4].

### Internal validation cohort

Five sets of duplicate samples were analyzed from the cancer cohort in order to examine for concordance.

### Statistical methods

#### Patient variables

Baseline patient characteristics were compared in the cancer and control arm to identify any differences in the study cohort. The continuous variables were analyzed using the unpaired student *t* test and presented as mean values with 95% confidence intervals. The categorical variables were analyzed using the chi-square test and presented as frequency percentages. A multivariate logistic regression analysis was then performed to identify significant co-variates for the breast cancer status.

#### Epigenetic variables

Despite high correlations, DNAmAge estimates can deviate substantially from chronological age at the individual level; by adjusting for chronological age, we can arrive at a measure of epigenetic age acceleration. DNA methylation age was regressed on chronological age (at the time of breast sample collection) using linear regression. Age acceleration was then defined as raw residuals resulting from the model. Thus, a positive or negative value indicates that a given breast sample is older or younger than expected based on chronological age, respectively. This measure of age acceleration is not correlated with chronological age (*r* = 0) and has a mean value of zero. All measures were calculated using a previously published online version of the DNAmAge calculator. We further calculated the mean methylation levels in the two groups. Pearson correlation statistic of methylation levels against age was calculated for cancer and control groups. Non-parametric tests were performed to test for mean differences in all the epigenetic variables within the two cohorts.

#### Regression models (univariate, multivariate, and IPWRA)

A linear regression model was plotted to define the collinearity of the DNAmAge with the age variable. The residuals from the plot were utilized to define the age acceleration residuals as mentioned before. A univariate and multivariate linear regression analysis was then performed to identify predictors of DNAmAge and age acceleration residuals. The *p* value < 0.05 was considered statistically significant. A regression adjustment model with inverse probability weighting (IPWRA) was created to address for the potential confounding variables. This treatment effects model was further bootstrapped for 500 repetitions to identify the 95% confidence intervals of the average treatment effect in population and the potential-outcome means. Average treatment effect in this model can be defined as the additional DNAmAge years of the tissue sample in breast cancer patients compared to controls in a matched population.

#### Predictive function of epigenetic variables—ROC curves

Receiver operating characteristic (ROC) were plotted for breast cancer status as the reference variable and age, DNAmAge, mean methylation by sample, age acceleration difference, and age acceleration residuals as classification variables. DeLong method was used to calculate the standard errors, and binomial confidence intervals were calculated. The ROC curves were plotted based on the binomial fit models, and the AUC was calculated. The sensitivity and specificity of the most predictive epigenetic variable was then calculated based on the ROC curve.

All the tables, graphs and statistical analysis was performed using STATA version 15.1 (StataCorp LLC, TX, USA). Original datasets used for statistical analysis are included as Additional files 1 and 2.

## Results

### Sample characteristics

Ninety-six normal breast tissue samples were obtained from 88 subjects (breast cancer = 35 subjects/40 samples, unaffected = 53 subjects/53 samples). Normal tissue samples from breast cancer patients were obtained from distant non-tumor sites of primary mastectomy specimens, while samples from unaffected women were obtained from the Komen Tissue Bank (*n* = 25) and from non-cancer related breast surgery specimens (*n* = 28). Three patients that received neoadjuvant chemotherapy in the cancer arm were excluded from analysis. Five additional samples were taken from specimens with breast cancer to serve as internal controls for studying any variations within the breast tissue. Samples from the breast cancer patients were classified as the “cancer arm,” and those from unaffected patients were classified as the “control arm.”

### Patient demographics

The baseline characteristics for the cancer arm and control arm have been summarized in Table 1**.** The mean age of patients was 49.7 years versus 45.9 years in the cancer arm and the control arm, respectively (*p* = 0.126). Most of the patients in our study cohort were Caucasian (86%) and non-Hispanic (91.4%). The average body mass index of the cancer group was 27 kg/m^2^. Forty percent of patients were ever-smokers, and 62% are current alcohol users. There was significantly higher alcohol consumption in the control group than the cancer group (72% vs 47%, *p* = 0.019). The patients were mostly premenopausal (60%), and 74% were ever-pregnant. The median live birth count was 2, and 41% patients had a history of breastfeeding. The mean age at menarche and mean age at first live birth were not significant between the two cohorts. Within the cancer cohort, patients were randomly distributed in terms of the pathological stage (0–III). Forty-five percent had a positive family history of breast cancer, and 95% of patients had ER+/PR+ tumors. Her2neu was positive in 7.5% of tumor samples, while 15% of patients were not typed for Her2neu. One patient in the control group was BRCA-positive, who had undergone a risk-reduction mastectomy. This patient was excluded from univariate and multivariate analyses. Further multivariate logistic regression analysis revealed that alcohol consumption and post-menopausal status was significantly different in the two cohorts. The details of the analysis have been summarized in Table 2.

**Table 1:** Demographic variables of the cancer and control arms

Variables	Breast cancer *N* (%)/mean (95% CI)	Controls *N* (%)/mean (95% CI)	*p* value
Total cohort samples	40	53	
Age (years)	49.7 (46.32–53.02)	45.9 (40.29–51.55)	0.126
Age category			0.742
< 50 years	24 (60%)	30 (57%)	
≥ 50 years	16 (40%)	23 (43%)	
Ethnicity			0.076
White	31(78%)	49 (92%)	
African Americans	4 (10%)	3 (6%)	
Others	5 (13%)	1 (2%)	
Ashkenazi Jew	6 (15%)	3 (6%)	0.162
Height (in.)	64.02 (63.10–64.94)	63.96 (63.19–64.73)	0.45
Weight (lbs)	157.22 (146.54–167.90)	157.50 (148.59–166.41)	0.483
BMI (kg/m^2)^			0.446
< 18.5	0 (0%)	1 (2%)	
18.5–24.9	21 (53%)	22 (42%)	
25.0–29.9	8 (20%)	17 (32%)	
> 30	11 (28%)	13 (25%)	
Tobacco use			0.696
No	25 (63%)	31 (58%)	
Yes	15 (38%)	22 (42%)	
Smoking (pack years)	3.73 (1.06–6.41)	3.86 (1.06–6.59)	0.475
Current alcohol use			0.019
No	20 (52%)	15 (28%)	
Yes	18 (47%)	38 (72%)	
Positive family history of breast cancer	18 (45%)	11 (21%)	0.012
Age at menarche (years)	12.37 (11.72–13.03)	12.54 (12.20–12.87)	0.671
Menopausal status			0.212
Pre-menopausal	27 (68%)	29 (55%)	
Post-menopausal	13 (33%)	24 (45%)	
Ever pregnant			0.266
No	8 (20%)	16 (30%)	
Yes	32 (80%)	37 (70%)	
No. of times pregnant	2.6 (1.94–3.25)	1.94 (1.48–2.39)	0.049
Age at first childbirth (years)	25.73 (23.24–28.99)	25.61 (24.21–27.01)	0.465
Number of live births	1.97 (1.54–2.40)	1.57 (1.23–1.92)	0.073
Breastfeeding			0.567
No	25 (63%)	30 (57%)	
Yes	15 (38%)	23 (43%)	
ER/PR status			NA
ER+/PR+	38 (95%)	–	
ER+/PR-	1 (2.5%)	–	
ER−/PR+	0(0)		
ER−/PR−	1(2.5%)	–	
Her2 status			NA
Not typed	6 (15%)	–	
Her−	31 (78%)	–	
Her+	3 (7.5%)	–

**Table 2:** Multivariate logistic regression predicting breast cancer

Logistic regression					Number of obs	57
					LR chi2(9)	25.9
					Prob > *χ*^2^	0.0021
Log likelihood			− 25.488985		Pseudo *R*^2^	0.3369
Breast cancer status	Odds ratio	Std. err.	*z*	*p* > *z*	[95% conf.	Interval]
Age	1.11	0.07	1.79	0.07	0.99	1.25
Age of first live birth	1.04	0.08	0.50	0.62	0.89	1.22
Age of menarche	1.33	0.38	0.99	0.32	0.76	2.34
Current alcohol intake	0.21	0.16	− 2.06	0.04	0.05	0.93
BMI	0.95	0.07	− 0.78	0.44	0.82	1.09
Ever breast fed	0.61	0.50	− 0.60	0.55	0.12	3.06
Family history	2.37	1.91	1.07	0.28	0.49	11.51
Post- vs pre-menopausal	0.01	0.02	− 2.60	0.01	0.00	0.32
Smoking (py)	0.89	0.08	− 1.29	0.20	0.74	1.06

### CpG methylation levels and the “epigenetic clock” analysis

**Fig. 1:**
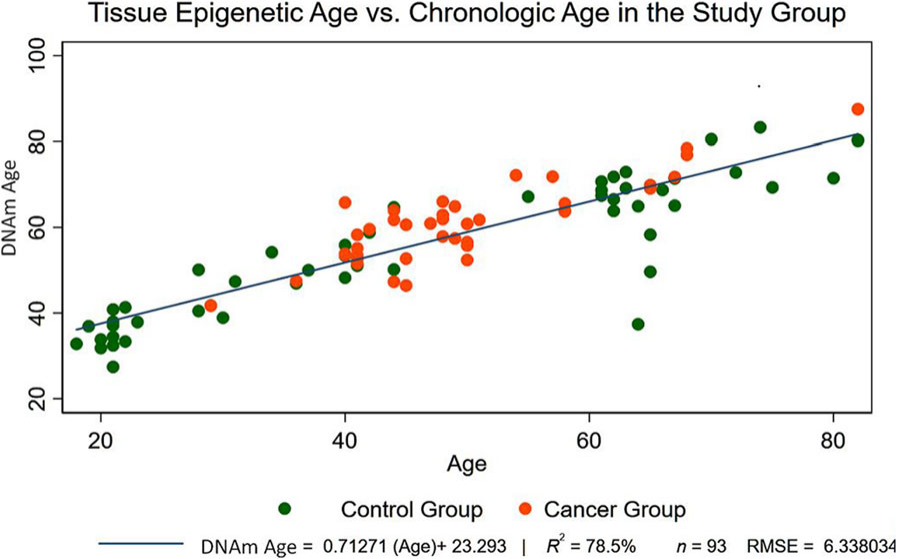
Tissue epigenetic age versus chronological age. DNA methylation age estimate based on 353 CpGs (*y*-axis) versus chronological age. All samples are normal breast tissue samples; normal tissue samples from cancer patients were obtained from mastectomy specimens > 3 cm from tumor margin. Samples (points) are colored by disease status of the donor: red = breast cancer and green = control. A linear regression line has been added. Age acceleration is defined as raw residual resulting from the regression model, i.e., the (signed) vertical distance to the line. Points above and below the line exhibit positive and negative epigenetic age acceleration, respectively. The high Pearson correlation coefficient *r* = 0.712, (*p* < 0.001) reflects the strong linear relationship between DNAmAge and chronological age at the time of breast sample collection

**Fig. 2:**
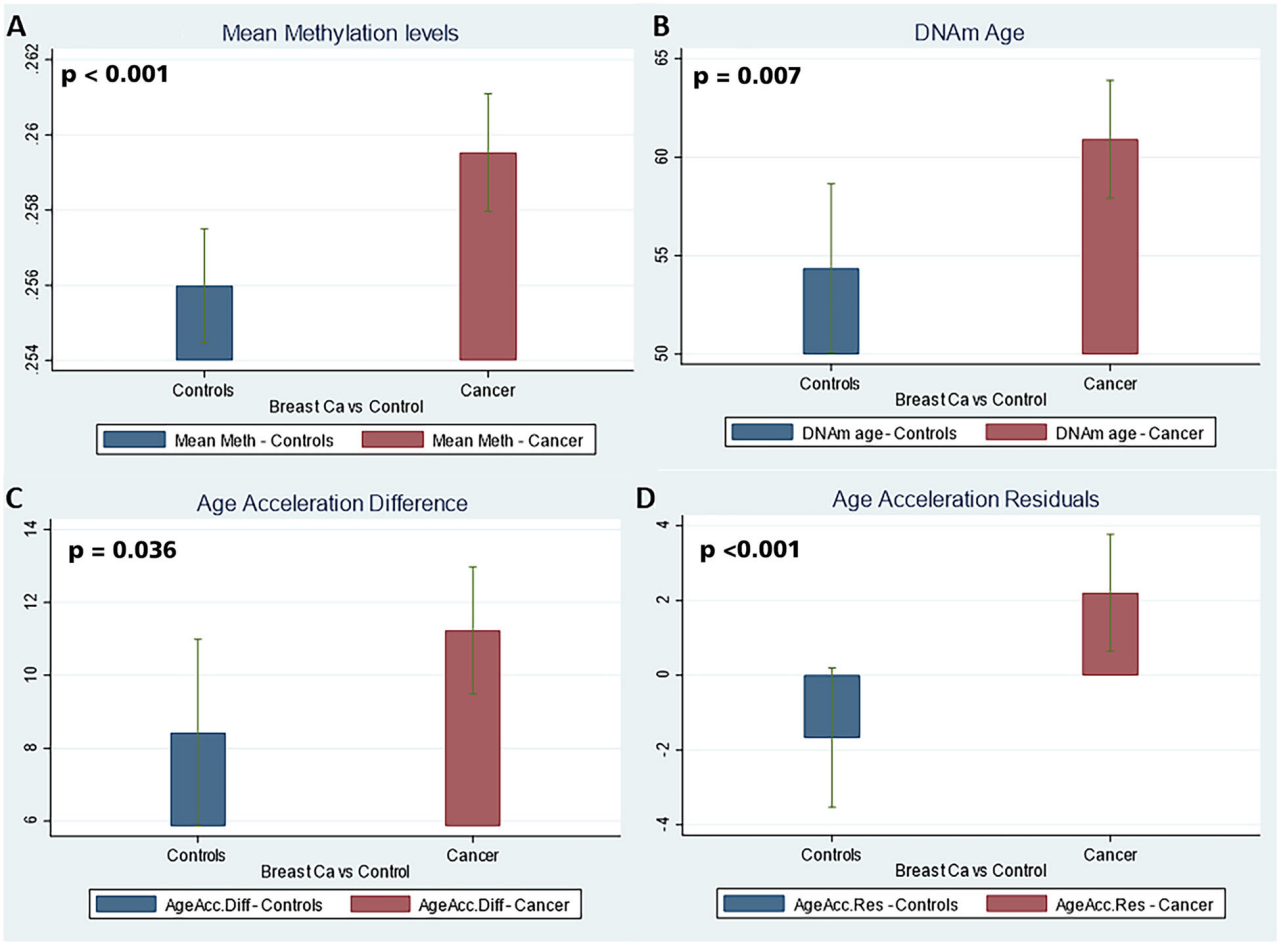
Comparison of epigenetic variables between cases and controls. Bar plots depict mean epigenetic age and age acceleration (*y*-axis) versus disease status. **a** Mean methylation levels of each cohort. **b** DNAmAge of each cohort. **c** Age acceleration differences. **d** Age acceleration residuals. The cancer cohort exhibits a significant positive age acceleration (positive residual coefficient) correlation compared to controls

The estimated DNAmAge (derived from the epigenetic clock) based on tissue CpG mean methylation levels highly correlated with the chronological age of the patients at the time of breast tissue collection (*r* = 0.712, *p* < 0.001, Spearman’s correlation test) (Fig. 1), (Table 3). This further confirmed the findings we had published previously that tissue-level methylation can serve as a predictor for the aging process in an individual [17]. Despite an increasing trend, it can be noted that the tissue epigenetic age varies widely for each individual. The cancer cohort showed a higher mean of DNAmAge than the control cohort on univariate analysis (*p* = 0.021, Student’s *t* test) and remained statistically significant even after matching for age and smoking status (*p* = 0.009). (Table 3) To eliminate the effect of age, we regressed the DNAmAge values over the age variable to calculate the age acceleration residuals. The cancer cohort exhibited a significant positive age acceleration (positive residual coefficient) correlation compared to the control samples (*p* < 0.001, Student’s *t* test) (Fig. 2d). All samples but one in the cancer cohort were ER+ and/or PR+ (luminal subtype). The single basal subtype did not show a positive age acceleration; however, no conclusion could be drawn from a single value. Three patients from the luminal subtypes were Her2+. Though these three patients had a positive age acceleration with respect to the controls, it was not significantly different from the Her2-negative cancer cohort (RR − 0.001, SE − 0.006, *p* = 0.237).

**Table 3:** Univariate and multivariate analyses of factors affecting DNAmAge and age acceleration residuals

	Univariate analysis			Multivariate analysis		
	Coef.	Std. err.	*p* > t	Coef.	Std. err.	*p* > *t*
DNAmAge						
*Age*	*0.712*	*0.039*	*< 0.001*	*0.807*	*0.095*	*< 0.001*
*Breast cancer vs controls*	*6.551*	*2.78*	*0.021*	*4.489*	*1.663*	*0.009*
BMI	0.148	0.238	0.534	0.164	0.143	0.256
Current alcohol use	− 2.833	2.904	0.332	–	–	–
*Smoking (py)*	*0.407*	*0.16*	*0.013*	*0.177*	*0.075*	*0.022*
Age at menarche	0.322	0.959	0.738	0.948	0.49	0.057
Age at first live birth	0.03	0.251	0.905	–	–	–
Count of live births						
1	2.292	5.494	0.678	− 4.92	3.268	0.137
1+	14.395	2.887	< 0.001	− 1.536	2.138	0.475
Breast fed	5.286	2.83	0.065	–	–	–
Post- vs pre-menopausal	18.645	2.138	< 0.001	− 3.982	3.006	0.19
Hispanic	− 5.924	5.018	0.241	− 3.921	3.223	0.228
Race						
White	− 0.059	5.815	0.992	0.709	3.088	0.819
African Americans	− 2.142	7.643	0.78	0.196	4.219	0.963
Age Acc. Residuals						
Age	0.000	0.039	1	0.095	0.095	0.324
*Breast cancer vs controls*	*3.878*	*1.264*	*0.003*	*4.489*	*1.664*	*0.009*
BMI	0.117	0.11	0.288	0.164	0.143	0.256
Current alcohol use	− 2.484	1.349	0.068	–	–	–
*Smoking (py)*	*0.174*	*0.074*	*0.022*	*0.178*	*0.076*	*0.022*
Age at menarche	0.358	0.454	0.433	0.949	0.491	0.057
Age at first live birth	− 0.169	0.153	0.274	–	–	–
Count of live births						
1	− 1.598	2.9	0.583	− 4.92	3.268	0.137
1+	0.041	1.52	0.979	− 1.536	2.138	0.475
Breast fed	− 2.28	1.315	0.086	–	–	–
Post vs pre-menopausal	− 1.351	1.335	0.314	− 3.982	3.006	0.19
Hispanic	0.636	2.342	0.786	− 3.921	3.223	0.228
Race						
White	− 1.063	2.685	0.693	0.709	3.088	0.819
African Americans	1.082	3.529	0.76	0.196	4.219	0.963

Variables in italics are those which reached statistical significance

### Predictors of DNAmAge and age acceleration residuals—univariate and multivariate analyses and inverse probability weighted regression adjustment (IPWRA) analysis

A univariate analysis revealed that age (*p* < 0.001), breast cancer status (*p* = 0.021), smoking (pack years) (*p* = 0.013), more than one live birth (*p* < 0.01), and post-menopausal status (*p* < 0.001) were significantly associated with DNAmAge. However, on the multivariate analysis only age (*p* < 0.001), breast cancer status (*p* = 0.009), and smoking pack years (*p* = 0.022) remained significant. Since DNAmAge has a very strong correlation with age of the individual (*r* = 0.713), it can be hypothesized that the effect of breast cancer status or any other covariate will be diminished. To adjust for this, we calculated an age-adjusted measure of DNAmAge as age acceleration residual, which is independent of the age of the patient (vide supra). This can be seen in Fig. 2 where age acceleration residual (*p* < 0.001) (Fig. 2d) has a stronger correlation than DNAmAge (*p* = 0.007). A multivariate analysis on age acceleration residuals revealed that only breast cancer status (*p* = 0.009) and smoking pack years (*p* = 0.022) were significant predictors of this epigenetic variable **(**Table 3**).** Further, breast cancer status is a much stronger predictor (coefficient = 4.489) of increased age acceleration residual than the smoking pack years (coefficient = 0.178) (Table 2**)**.

**Fig. 3:**
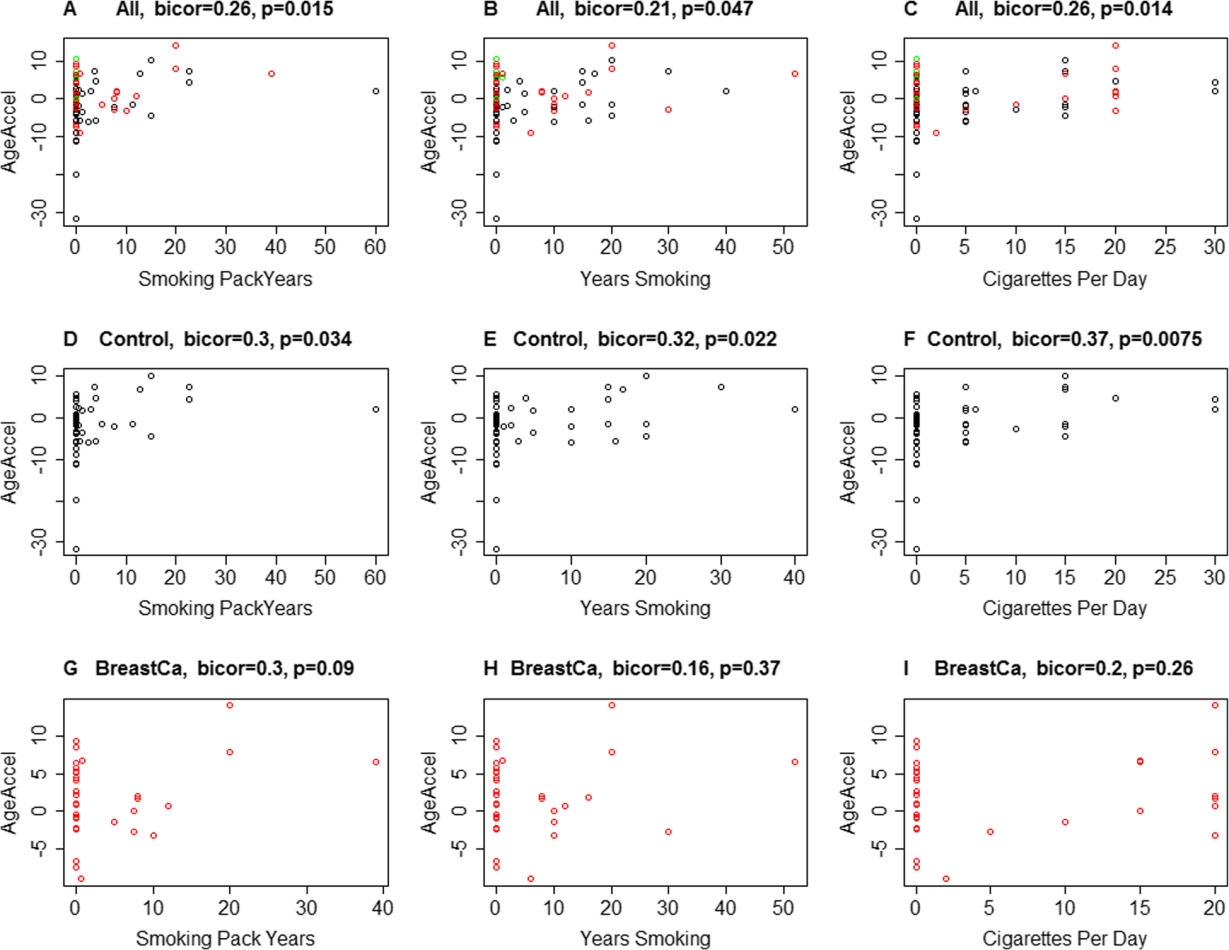
Correlation of tobacco use variables between cases and controls. DNA methylation age acceleration estimates (*y*-axis) are depicted for specific tobacco use variables, including total pack years, total number of years smoking, and cigarettes per day. **a**–**c** Analyses for these variables using the combination of both study cohorts. **d**–**f** The same specific variable analyses for the control cohort. **g**–**i** Analyses for the cancer cohort. There is a statistically significant positive correlation between the tobacco variables and the complete cohort, and in the control cases. Though a positive correlation is noted in breast cancer cases as well, it did not reach a statistical significance

In a secondary analysis, we examined the correlation of smoking pack years with the DNAmAge of the sampled breast tissue. Age acceleration residual was correlated with tobacco variables (*r* = 0.21, *p* = 0.047 for total years of smoking, *r* = 0.26, *p* = 0.014 cigarettes per day, and *r* = 0.26, *p* = 0.015 smoking pack years) in the complete cohort. Similar trends were also noted in the control group (Fig. 3a–f). Though a positive correlation was also noted in the cancer group, it did not reach statistical significance (Fig. 3g–i). These results need to be interpreted with caution as the study was not designed initially to identify smoking as a potential driver of tissue-level epigenetic changes.

Our study population had a selection bias in the age of presentation of the cancer and control population. This can be identified in Fig. 1, where most of the patients in the cancer cohort were in the age group 45 to 65 years whereas the control group were either below or above this age group. To adjust for this, we created an IPWRA model based on predictors of DNAmAge (linear-dependent outcome variable) and breast cancer status (logistic-dependent treatment variable), accounting for age and smoking as independent outcome variables and current alcohol use and menstrual status as independent treatment variables, to identify the average treatment effect. The iterations were further bootstrapped to 500 reps to calculate the 95% CI. The analysis revealed that breast cancer status was significantly associated with a higher DNAmAge score with an average treatment effect of 3.98 years (*p* = 0.003) (Table 4).

**Table 4:** Regression adjustment model with inverse showing average treatment effects (ATE) and potential-outcome mean (POmean)

DNAmAge	Groups	Coef.	Bootstrap	*z*	*p* > *z*	[95% conf. interval]	
			Std. err.				
ATE	Cancer vs control group	3.98337	1.333459	2.99	0.003	1.369837	6.596902
POmean	Control	55.60416	1.642661	33.85	0	52.38461	58.82372

Treatment-effects estimation: Number of obs = 85; Estimator: IPW regression adjustment; Outcome model: linear; Treatment model: logit; Bootstrap Iterations: 500

### Predictive function of the epigenetic variables

**Fig. 4:**
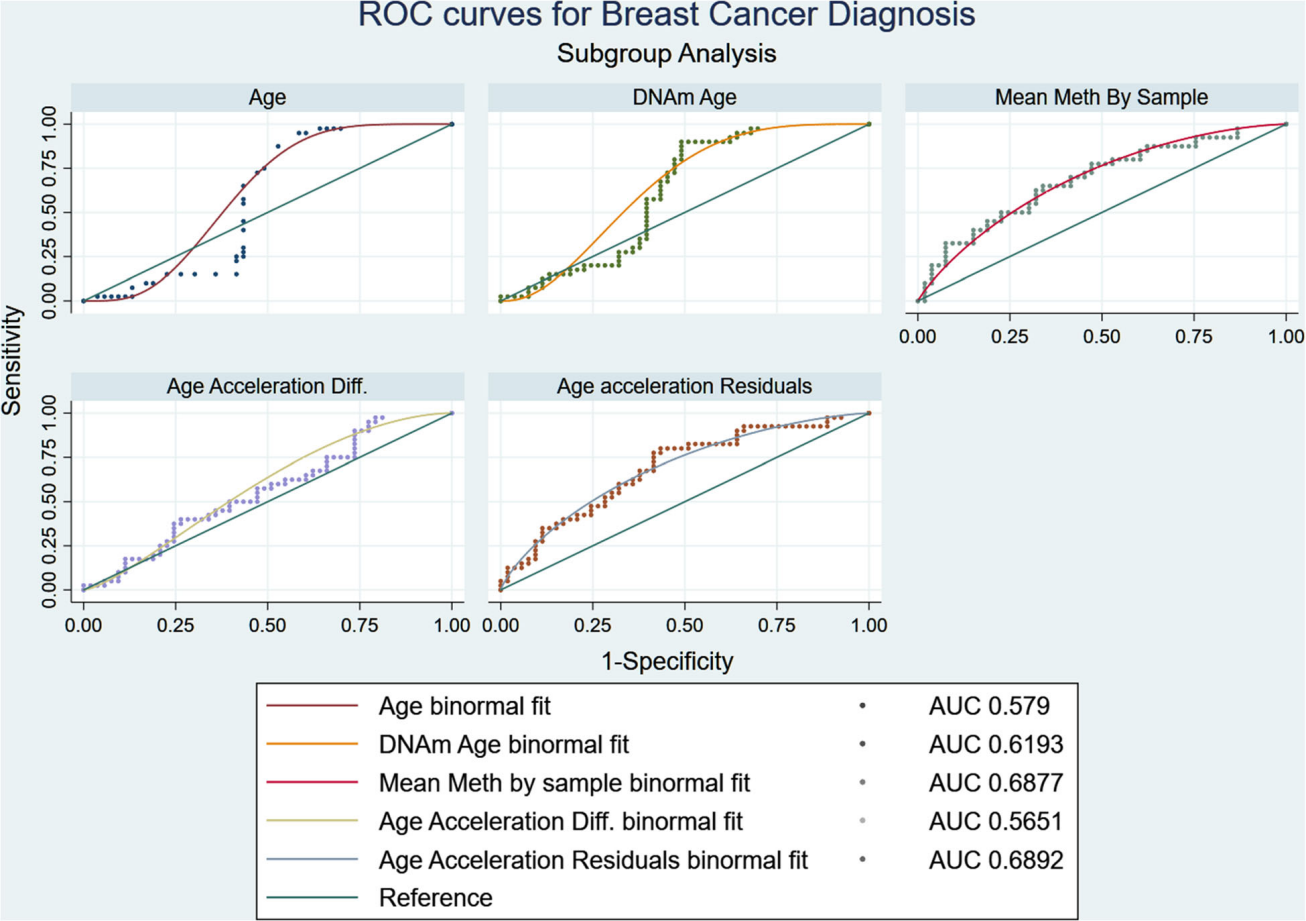
Receiver operating characteristic for all epigenetic variables. Receiver operating characteristic (ROC) were plotted for breast cancer status as the reference variable and age, DNAmAge, mean methylation by sample, age acceleration difference, and age acceleration residuals as classification variables. DeLong method was used to calculate the standard errors, and binomial confidence intervals were calculated. The ROC curves were plotted based on the binomial fit models, and the AUC was calculated. The sensitivity and specificity of the most predictive epigenetic variable was then calculated based on the ROC curve

The receiver operating characteristic (ROC) were plotted to identify the accuracy of the epigenetic variables in predicting breast cancer (Fig. 4). Age is considered a strong risk factor for breast cancer; thus, its ROC curve was considered as the baseline for comparison (AUC = 0.527). Like age, DNAmAge (AUC = 0.578) was not a good predictor for breast cancer status, and the curve closely resembled the age binomial fit model. This can be attributed to age being a stronger predictor for DNAmAge than breast cancer. Age difference calculated as the difference of the epigenetic age from the chronological age did not reach desired predictive accuracy as well.

Both mean methylation by sample and age acceleration residuals lead to ROC curves that lie above the reference line (AUC = 0.687 and AUC = 0.689, respectively) (Fig. 4). The mean age acceleration for the control cohort was − 1.67 which corresponded to a sensitivity of 82.5% and specificity of 49.06% with a positive likelihood ratio 1.62 and negative likelihood ratio 0.35. The mean age acceleration residual for cancer cohort was 2.21 which corresponded to a sensitivity of 47.50% and specificity of 75.47% with a positive likelihood ratio 1.94 and negative likelihood ratio 0.69. The tradeoff was achieved at − 0.920 with 80% sensitivity and 57% specificity.

## Discussion

To our knowledge, this is the first study that has analyzed the epigenetic age variables of the adjacent “normal” breast tissue in patients with breast cancer. Our cross-sectional analysis suggests that both epigenetic age acceleration and mean methylation in adjacent breast tissue are predictive of breast cancer status, but these findings require validation in prospective cohort studies. Age, along with breast cancer status and smoking, are independent predictors of epigenetic age of breast tissue.

It is unknown what age-related genetic changes come in effect to increase the incidence of breast cancer in the age group 45–65 years and whether the changes are limited to the site of tumor origin or are present in the entire breast tissue. The concept of field cancerization is well-known in other regions of the body where it has been attributed to exposure to exogenous factors; however, the role of endogenous factors like chronic cell cycling or age-related epigenetic silencing of various genetic pathways in making a tissue more vulnerable to oncogenic transformation is not fully identified. Certain aging processes can accelerate or hinder tumorigenesis in a tissue-specific manner which has been discussed elsewhere [18]. Its specific role in the breast cancer is yet to be elucidated.

Our study highlights the treatment effects analysis which suggests that the normal tissue in the breast cancer patients was at least half a decade older in terms of cumulative epigenetic damage in an age-matched comparison. While this finding may initially seem to have little clinical significance, it is interesting to note that the age acceleration residuals were in complete contrast within the two cohorts. Unaffected individuals had a negative mean age acceleration residual, suggesting that the rate of increase of breast tissue age was slowing down in terms of chronological age, compared to the patients with breast cancer who had positive age acceleration residual, suggesting that the breast tissue was aging at a faster rate than the individual herself. The ROC curves further suggest that higher age acceleration in the breast cancer cohort was specific for breast cancer occurrence. Although our cross-sectional model does not lend itself for dissecting cause and effect relationships, the significant age acceleration observed in patients with luminal breast cancers supports the hypothesis that DNAmAge of normal breast tissue in women with breast cancer increases at a higher rate than in an unaffected individual. As such, our findings suggest that a breast tissue biomarker of accelerated aging may exist that could potentially be associated with the future development of breast cancer.

Future studies will need to test the hypothesis that breast tissue is more predictive of incident breast cancer than blood tissue, which has previously been shown to have a positive, but relatively weak, predictive association [14]. This hypothesis is indirectly supported by the finding that DNA methylation levels in breast tissue are more predictive of the endogenous hormonal milieu in unaffected women compared to blood [19]. Thus, it will be interesting to study whether DNA methylation changes precede actual occurrence of the breast cancer in patients with hormone-responsive breast cancers.

The positive association of smoking with the DNAmAge as well as age acceleration residual is an interesting and unexpected finding in our study. Previous studies failed to detect such an effect in blood [20], liver, or adipose tissue [10]. Taken together, these findings corroborate the hypothesis that many stress factors affect epigenetic age acceleration in a tissue-specific manner. Ever-smokers have been found to have a modest increase in the incidence of breast cancer, particularly in females who started smoking in their adolescence. Although our study does not include data on the exact time interval since smoking initiation and/or smoking cessation and acquisition of data, the association of an overall impact of smoking on DNA methylation is intriguing and merits further study.

We recently published our findings that breast tissue ages faster than blood in unaffected women, as measured by DNA methylation [17]. From the current study, we further extend our understanding of the normal breast tissue, where we identify that patients with hormone-responsive breast cancer have higher epigenetic age acceleration compared to age-matched controls. Given that breast tissue age could be considered a function of multiple variables orchestrating in sync in response to endogenous and exogenous factors during an individual’s lifetime (such as age of menarche, use of hormone replacement therapy, alcohol use, and others), epigenetic aging may serve as a useful surrogate marker of this changing internal milieu, and offer insight into future breast cancer risk.

We acknowledge the limitations of our study, including the aforementioned small sample size and inability to extrapolate findings to all breast cancer subtypes. Our study involved mainly luminal breast cancer samples. We had one ER-negative/PR-negative sample (which exhibited negative age acceleration) and one ER+/PR-negative sample, and thus, no definite conclusions could be drawn from them. We were also not able to evaluate the effect of BRCA mutations on epigenetic age acceleration since our study involved only a single BRCA mutation carrier who had not (yet) developed breast cancer at the time of sample collection. Further, no significant correlation could be drawn based on the pathologic stage of the tumor as the sample size was not powered for such an analysis. An additional limitation of our study was the difference in age distribution of the cancer cohort as compared to the unaffected cohort, though statistical measures were taken to account for this difference. Future studies with closely age-matched cohorts would be helpful to corroborate our findings. Finally, and of note, we did not isolate any specific cell type within the whole breast sample for the epigenetic age analysis. Thus, we cannot account for the specific cell type, if any, that is primarily responsible for the DNAmAge acceleration in the normal breast. Future studies should be considered to determine the epigenetic ages of individual cell types, as compared to whole tissue epigenetic age analysis.

## Conclusions

In summary, our study demonstrates that epigenetic age acceleration of the “normal” breast tissue in patients with luminal breast cancer was significantly higher than that of unaffected women. We also observed that the difference was maintained when adjusted for potential clinical confounders. Further larger prospective studies will be required to identify the temporal trend of the observed epigenetic aging and its possible use as a predictive biomarker.

## Additional files

Additional file 1: Subject clinical datafile. (XLSX 25 kb)

Additional file 2: Raw statistical datafile. (XLSX 27 kb)

## Abbreviations

AUC: Area under the curve
CI: Confidence interval
CpGs: Cytosine-phosphate-guanines
DNAmAge: DNA methylation age
ER: Estrogen receptor
IPWRA: Inverse probability weighted regression adjustment
KTB: Komen Tissue Bank
PR: Progesterone receptor
ROC: Receiver operating characteristic
